# Does Pre-Emptive Availability of PREDICT 2.1 Results Change Ordering Practices for Oncotype DX? A Multi-Center Prospective Cohort Study

**DOI:** 10.3390/curroncol31030096

**Published:** 2024-02-27

**Authors:** Arif Ali Awan, Deanna Saunders, Gregory Pond, Caroline Hamm, Nadia Califaretti, Mihaela Mates, Vikaash Kumar, Mohammed F. K. Ibrahim, Ana-Alicia Beltran-Bless, Lisa Vandermeer, John Hilton, Mark Clemons

**Affiliations:** 1 Division of Medical Oncology and Department of Medicine, University of Ottawa, Ottawa, ON K1H 8M5, Canada; aawan@ohri.ca (A.A.A.); abeltran@toh.ca (A.-A.B.-B.); jfhilton@toh.ca (J.H.); 2 Cancer Therapeutics Program, Ottawa Hospital Research Institute and Ottawa Hospital, Ottawa, ON K1H 8L6, Canada; 3 Department of Oncology, McMaster University, Hamilton, ON L8S 4L8, Canada; 4 Windsor Regional Hospital, Windsor, ON N9A 1E1, Canada; caroline.hamm@wrh.on.ca; 5 Grand River Regional Cancer Centre, Kitchener, ON N2G 1G3, Canada; nadia.califaretti@grhosp.on.ca; 6 Kingston Health Sciences Centre, Kingston, ON K7L 5G2, Canada; mihaela.mates@kingstonhsc.ca; 7 Markham Stouffville Hospital, Markham, ON L3P 7P3, Canada; vikaash.kumar@uhn.ca; 8 Division of Oncology, Northern Ontario School of Medicine, Sudbury, ON P3E 2C6, Canada; mohammed.ibrahim@tbh.net

**Keywords:** PREDICT 2.1, Oncotype DX, early-stage HR+ breast cancer, real-world evidence

## Abstract

For early-stage hormone receptor (HR)-positive and HER2-negative breast cancer, tools to estimate treatment benefit include free and publicly available algorithms (e.g., PREDICT 2.1) and expensive molecular assays (e.g., Oncotype DX). There remains a need to identify patients who de-rive the most benefit from molecular assays and where this test may be of poor value. In this multicenter prospective cohort study, we evaluated whether use of PREDICT 2.1 would impact physician decision making. For the first 6 months of the study, data on physician use of both PREDICT 2.1 and Oncotype DX ordering were collected on all newly diagnosed patients eligible for molecular testing. After 6 months, an educational intervention was undertaken to see if providing physicians with PREDICT 2.1 results affects the frequency of Oncotype DX requests. A total of 602 patients across six cancer centers in Ontario, Canada were recruited between March 2020 and November 2021. Providing PREDICT 2.1 results and an educational intervention did not alter the ordering of an Oncotype DX. For patients with low clinical risk, either by clinico-pathologic features or by PREDICT 2.1, the probability of obtaining a high Oncotype DX recurrence score was substantially lower compared to patients with high-clinical-risk disease. The introduction of an educational intervention had no impact on molecular assay requests. However, routine ordering of molecular assays for patients with low-clinical-risk disease is of poor value.

## 1. Introduction

Multiple prognostic and predictive tools have been developed to guide the care of patients with newly diagnosed early-stage hormone receptor (HR)-positive and human epidermal growth factor receptor (HER2)-negative breast cancer [1,2,3,4]. These include free and publicly available algorithms (e.g., PREDICT 2.1 [5], Magee equations (1,2,3) [6,7], Gage [8], and University of Tennessee probability models [9]) and IHC4 that incorporates immuno-histochemical markers to more expensive molecular tests such as Oncotype DX, MammaPrint, PAM50, EndoPredict, and Breast Cancer Index [10,11,12,13,14,15].

The PREDICT 2.1 model is an online tool available at https://breast.predict.nhs.uk/tool (accessed on 22 February 2024) [16]. It uses readily available clinico-pathologic characteristics including age at diagnosis, number of lymph nodes involved, tumor size, histological grade, ER/Her2/Ki67 status, mode of detection (screening vs. clinical), and type of adjuvant systemic therapy (chemotherapy, trastuzumab, endocrine therapy, and bisphosphonate) [5]. The results of meta-analyses from Early Breast Cancer Trialists’ Collaborative Group (EBCTCG) from different systemic therapy modalities provide an estimate of an individual patient’s overall survival (OS) at 5, 10, and 15 years with different adjuvant systemic treatments. Although the recent version of the PREDICT model better predicts mortality for younger patients with breast cancer, there are limitations as it was developed with data prior to some contemporary chemotherapy regimens and uses population outcomes to generate individual risk/benefit estimates [16].

In contrast, the Oncotype DX Recurrence Score (Exact Sciences) is a widely used prospectively validated molecular prognostic and predictive test used to guide treatment in patients with early-stage HR-positive disease. Oncotype DX is a 21-gene expression assay of 16 known cancer-related genes and 5 reference genes, which provides a recurrence score (0 to 100) using RNA extracted from formalin-fixed, paraffin-embedded tissue [13]. While Oncotype DX use reduces the overall use of adjuvant chemotherapy, its use presents challenges. First, testing is costly (USD~4000), restricting its use to resource-rich countries. Second, while Oncotype DX use can reduce the odds of chemotherapy in node-positive or large node-negative disease, paradoxically, it can increase the odds of receiving chemotherapy in patients with small node-negative cancers, likely due to overestimation of clinical risk and treatment benefit [17,18]. To overcome this limitation, recently, pathological and clinical features such as tumor size, grade, and patient age were incorporated with the RS in the RS-pathology-clinical (RSPC) tool, which better predicts distant recurrence than RS alone [19]. More recently, the RSPC tool has been updated with additional larger and more contemporary data to become the RSClin tool and provide a more individualized estimate of incremental absolute chemotherapy benefit [20]. Alternatively, low clinical risk has also been defined in clinical studies as ER-positive and HER2-negative cancers that are ≤3 cm and grade 1, ≤2 cm and grade 2, and ≤1 cm and grade 3 [21].

There is notable variation in the ordering of molecular assays among physicians, practice settings, and countries as well as a need to identify patients who will derive the most clinical and economic benefit from testing. In this prospective cohort trial, we will evaluate whether the pre-emptive availability of PREDICT 2.1 results for individual patients impacts physician decision making with respect to ordering Oncotype DX. These data will also be used to provide further validation of the algorithms in a real-world, multi-center setting.

## 2. Materials and Methods

**Figure 1:**
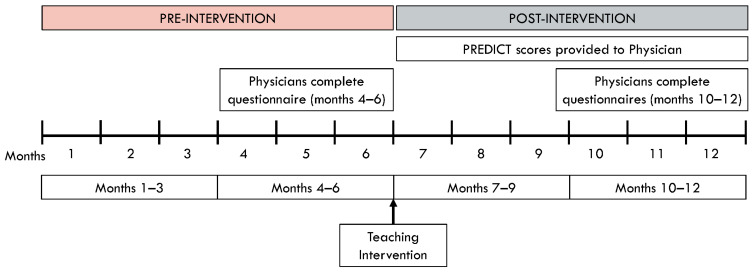
Study Schema: months 0–3: clinical data captured without any intervention; months 3–6: physicians provided with questionnaire; at the 6-month mark: educational intervention performed; months 7–9: physicians provided with PREDICT 2.1 results; months 10–12: physicians provided with PREDICT 2.1 results and questionnaire.

This is a multi-center prospective cohort study, with a pre- versus post-educational intervention comparison, evaluating if making individual patient PREDICT 2.1 results readily available to treating physicians impacts the ordering of Oncotype DX, chemotherapy use, and time to commencement of chemotherapy (Figure 1). In addition, to understand physician reasoning and comfort with making systemic therapy decisions with either PREDICT 2.1 or Oncotype DX or both, a physician questionnaire was also performed. The study was approved by the Ontario Cancer Research Ethics Board (OCREB ID 1952). Deliberately, no patient or physician consent was required due to concerns regarding biased results and non-applicability to broader clinical practice (Supplementary File S1: study protocol).

The study included patients with newly diagnosed early-stage, lymph node-negative breast cancer who were eligible for Oncotype DX testing under the established funding criteria in Ontario, Canada. These parameters were ER-positive, PR-positive or -negative, HER2-negative, lymph node status-negative or micro-invasive disease, and tumor >1 cm in size (or if ≤1 cm, must be grade 2 or 3 or have lymph node micrometastasis). Patients with neoadjuvant treatment and recurrent breast cancers were excluded.

Patients entered study screening when a complete pathology report was received. Once deemed eligible, they were assigned a Study ID number. Patients’ clinical, pathological, and adjuvant treatment data were obtained from their chart. During the first 3 months of the study, the usual practice pattern of the site was captured (Months 0–3). After 3 months of activation, physicians were asked to complete a questionnaire (Supplementary File S2: physician questionnaire) for each new patient with an eligible sample (Months 4–6). After 6 months of activation, the physicians received an educational session (Intervention) consisting of PowerPoint slides providing updated review on the benefits and limitations of Oncotype DX and PREDICT 2.1 testing (Supplementary File S3: educational intervention). After this session, staff at each site provided PREDICT 2.1 results for each patient to the treating physician prior to their meeting with each patient (Months 7–9). Again after 3 months, the physicians continued to receive PREDICT 2.1 results and the physician questionnaire was re-introduced (Months 10–12) (Figure 1).

Our primary hypothesis was that following the educational intervention, there would be a 50% reduction in Oncotype DX assay requests if physicians were pre-emptively provided with PREDICT 2.1 risk/benefit estimates. Secondary endpoints, identified as time from surgery to starting chemotherapy, type of chemotherapy, and number of patients receiving chemotherapy were also calculated. Results are presented using descriptive statistics for each time period separately. Results were compared between the pre-intervention phase and post-intervention phase using Fisher’s exact tests and Wilcoxon rank sum tests for dichotomous and continuous variables. Some category variables were dichotomous for statistical power purposes. No statistical adjustments were performed for multiple testing, and exact *p*-values are provided. All tests were two-sided, and statistical significance was defined at the α = 0.05 level; however, given the descriptive nature of this study, any statistically significant result for secondary outcomes must be interpreted with caution.

## 3. Results

### 3.1. Sites and Patients

The study was open March 2020–November 2021. All study sites were cancer centers in Ontario. Each site was open for one year with 321 patients accrued during the pre-intervention period and 281 patients accrued in the post-intervention period (Table 1).

### 3.2. Patient Characteristics

The clinical and pathologic characteristics are listed in Table 1. The mean age was 63.2 years, and 79% of patients were post-menopausal. The median size of the tumors was 16.5 mm with approximately 60% (370/602) having grade 2 cancers and 4% (25/602) having lymph node micrometastases.

### 3.3. PREDICT 2.1 Results

PREDICT 2.1 testing results are reported in Table 2. The estimated 10-year OS for these patients with early-stage HR-positive and HER2-negative without macrometastasis in the lymph nodes ranged from 72.6 to 76.1% with an additional OS benefit of 1.20–1.24% at 10 years from the addition of 2--generation chemotherapy. Based on the PREDICT 2.1 10-year OS benefit from 2nd generation chemotherapy, 57% (343/602) of patients had up to a 1% benefit, whereas 5.8% (35/602) of patients had a >3% benefit. Approximately 2/3 (379/602) and 1/3 (223/602) were classified as having a low clinical (defined as having cancers that are ≤3 cm and grade 1, ≤2 cm and grade 2, and ≤1 cm and grade 3) and high clinical (defined as cancers not meeting the low-risk criteria) risk, respectively.

### 3.4. Effect of Educational Intervention on Oncotype DX Requests

The educational intervention did not reduce Oncotype DX assay requests by the pre-specified criteria of 50% (48.0% (154/321) pre-intervention vs. 44.1% (124/281) post-intervention) (Table 3). With regard to secondary endpoints, the time from resection surgery to starting chemotherapy, type of chemotherapy, number of patients receiving chemotherapy, and number of patients receiving endocrine therapy did not change significantly pre- and post-educational intervention.

### 3.5. Oncotype DX Ordering and Oncotype DX Recurrence Score of ≥26 Depending on Clinical Risk

**Figure 2:**
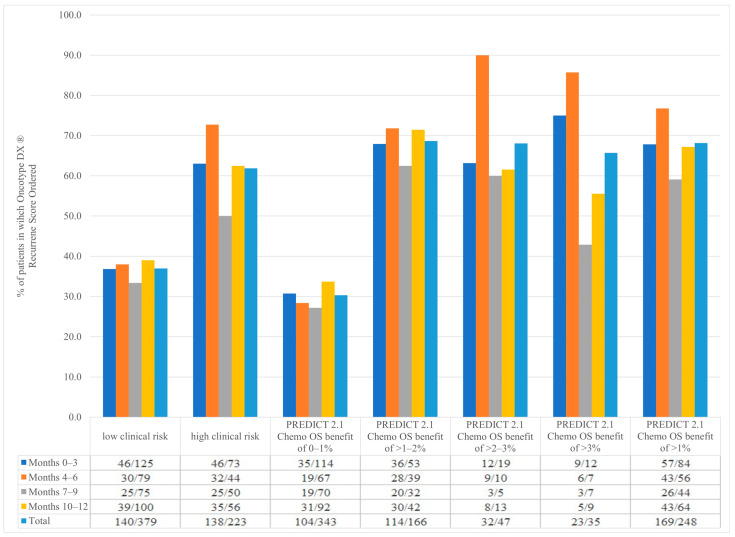
Patients with Oncotype DX^®^ ordered depending on clinical risk, PREDICT 2.1 2nd generation chemotherapy overall survival benefit through months 0–3, 4–6, 7–9, and 10–12 in different stages of the study.

In this study, 379 patients were classified as having low clinical risk. Within this sub-population, 36.9% (140/379) had an Oncotype DX requested. In contrast, in the 223 patients classified as having high clinical risk, 61.9% (138/223) had an Oncotype DX ordered (Figure 2).

**Figure 3:**
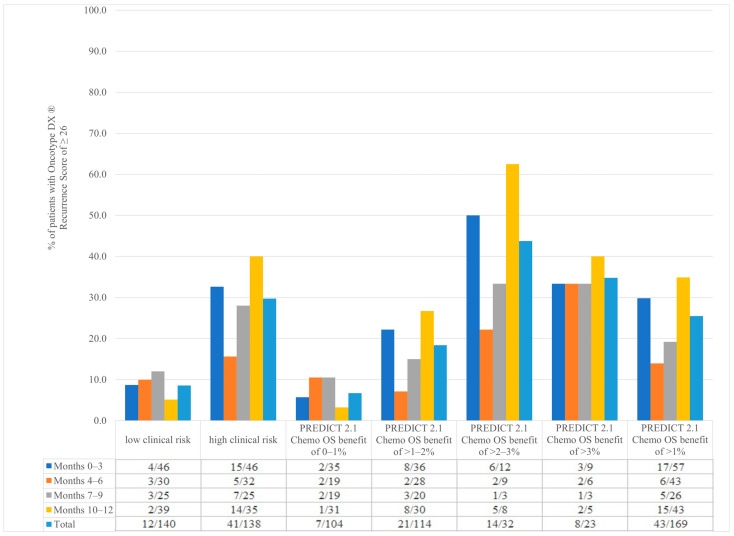
Patients with Oncotype DX^®^ recurrence score of ≥26 depending on clinical risk, PREDICT 2.1 2nd generation chemotherapy overall survival benefit through months 0–3, 4–6, 7–9, and 10–12 in different stages of the study.

Our data found a difference in the frequency of having an Oncotype DX recurrence score ≥ 26 between low-clinical-risk and high-clinical-risk populations. In patients with low-clinical-risk disease who had an Oncotype DX ordered, only 8.6% (12/140) had an Oncotype DX recurrence score ≥ 26 compared to 29.7% (41/138) of patients with high clinical risk (Figure 3). This means that for patients with low clinical risk, an Oncotype DX assay will need to be ordered on 11.6 patients before identifying one with a score ≥ 26. In contrast, patients with high-clinical-risk disease, an Oncotype DX assay will need to be ordered on 3.4 patients before identifying one with a score ≥ 26.

### 3.6. Oncotype DX Ordering and Oncotype DX Recurrence Score of ≥26 Depending on PREDICT 2.1 Results

In our study, 343 patients had a PREDICT 2.1 10-year OS benefit from 2nd generation chemotherapy of 0–1%. Despite pre-emptive notification of low clinical benefit based on PREDICT 2.1 scores (absolute 2nd generation chemotherapy benefit < 3%), 30.3% (104/343) of these patients still had an Oncotype DX ordered. The frequency of the Oncotype DX assay did increase with increasing estimated chemotherapy benefit (68.7% of patients’ tests if estimated benefit 1–2%; 68.0% if estimated benefit 2–3%, and 65.7% if estimated benefit > 3%). Of note, of the 602 total patients enrolled, only 35 (5.8%) were estimated by PREDICT 2.1 to have an absolute benefit of >3% from chemotherapy (Figure 2).

Similarly, the frequency of Oncotype DX recurrence scores ≥ 26 increased with increasing PREDICT 2.1-estimated absolute 2nd generation chemotherapy benefit. In patients with a PREDICT 2.1 chemotherapy OS benefit of 0–1% who had an Oncotype DX ordered, 6.7% (7/104) had an Oncotype DX recurrence score of ≥26 compared to 25.4% (43/169) of patients with a PREDICT 2.1 chemotherapy OS benefit of greater than 1% (Figure 3). In other words, for patients with a PREDICT 2.1-estimated chemotherapy benefit of 0–1%, 14.9 patients will need to be tested to identify one patient with an Oncotype DX recurrence score of ≥26. For patients with a PREDICT 2.1-estimated chemotherapy benefit of >1%, 3.9 patients will need to be tested to identify one patient with an Oncotype DX score of ≥26.

### 3.7. Comparison of RSClin Scores with Clinical Risk and PREDICT 2.1 Results for Benefit from 2nd Generation Chemotherapy

**Figure 4:**
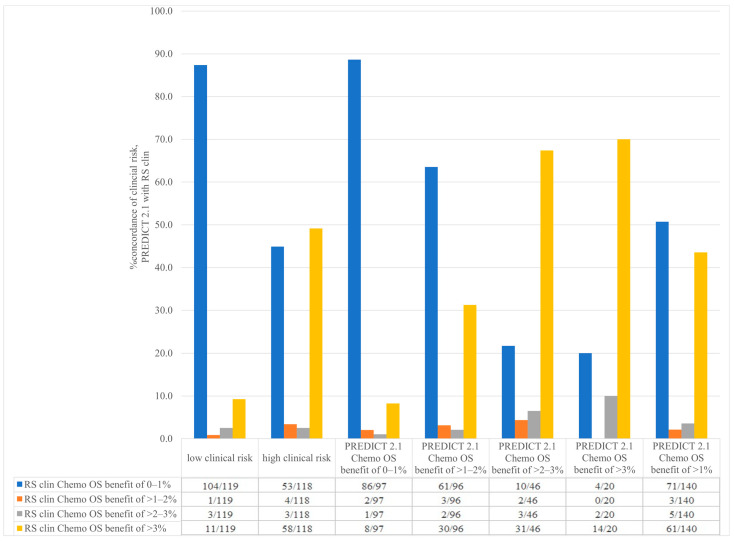
Concordance between RSclin-individualized absolute chemotherapy benefit depending on clinical risk and PREDICT 2.1 2nd generation chemotherapy overall survival benefit.

In patients with low clinical risk (absolute 2nd generation chemotherapy benefit of <3%), 9.2% (11/119) had an RSClin-individualized absolute chemotherapy benefit of greater than 3% compared to 50.8% (60/118) of patients with high clinical risk who had an RSClin-individualized absolute chemotherapy benefit of 0–3% (Figure 4). This implies that an Oncotype DX would estimate a greater than 3% absolute chemotherapy benefit in one in every 10.9 patients with low-clinical-risk disease and avoid chemotherapy in one in every two patients with high clinical disease if 3% is used as an arbitrary cut-off for recommendation of chemotherapy. Comparing PREDICT 2.1 10-year 2nd generation chemotherapy OS benefit and RSClin-individualized absolute chemotherapy benefit, we found 88.7% (86/97) of patients with a PREDICT 2.1 chemotherapy OS benefit of 0–1% also had an RSClin-individualized chemotherapy benefit of 0–1% (Figure 4).

### 3.8. Physician Questionnaires

We found that prior to the PREDICT 2.1 results being made routinely available, 52.5% of clinicians were already using it independent of the study as part of their routine standard of care (Table 4). Use increased to 86.5% after the PREDICT 2.1 results were made available to the clinicians in clinics. Most clinicians found that using PREDICT 2.1 allowed them to be more confident in their recommendations, provide additional clinically relevant information, and influence their treatment recommendations, and they would use the tool again.

## 4. Discussion

In this prospective cohort study, we found that providing PREDICT 2.1 results and slides highlighting the benefits and limitations of both PREDICT 2.1 and Oncotype DX did not reduce the frequency of ordering an Oncotype DX. In addition, use of PREDICT 2.1 did not improve time from resection to starting chemotherapy or change type of chemotherapy or number of patients receiving chemotherapy. These observations were contrary to our hypothesis that by clearly identifying clinical low- and high-risk (either through clinical risk or PREDICT 2.1 with absolute 2nd generation chemotherapy benefit of <3%) patients at baseline prior to assessment, we could reduce the need for use of molecular assays in the clinic and treat patients sooner on appropriate anti-cancer therapy.

Nevertheless, we did observe that the probability of obtaining an Oncotype DX recurrence score of ≥26 seems to be associated with both clinical risk and PREDICT 2.1-estimated risk. For example, for patients with low clinical risk, the probability of obtaining a high Oncotype DX recurrence score was substantially lower compared to patients with high-clinical-risk disease (1/11.6 patients vs. 1/3.4 patients, respectively). Similar findings were observed when PREDICT 2.1 was used to estimate clinical risk and chemotherapy benefit; in patients with a predicted chemotherapy OS benefit of 0–1% who had an Oncotype DX ordered, only 6.7% had a recurrence score ≥26 compared to 25.4% when PREDICT 2.1 predicted a chemotherapy OS benefit >1%. These observations associate well with the commercial RSClin score, where 88.7% (86/97) of patients with a PREDICT 2.1-estimated chemotherapy OS benefit of 0–1% also had an RSClin-individualized chemotherapy benefit of 0–1%.

Our data raise an important issue. For patients with low-clinical-risk disease or those with a PREDICT 2.1 2nd generation 10-year OS benefit of 0–1%, the probability of obtaining a recurrence score ≥ 26 if one orders an Oncotype DX is low. In addition, although molecular risk remains important in guiding patients in their decision making for adjuvant chemotherapy, there is a lack of clear evidence that patients with low-clinical-risk disease derive substantial benefit from chemotherapy even if their molecular risk is high. To our knowledge, the only study that looked at this in a randomized prospective manner is MINDACT (using MammaPrint), where approximately 345 patients in each arm with low clinical risk and high genomic risk were randomized to chemotherapy or no chemotherapy. In this study, there was no evidence of benefit to OS, survival without distant metastases, and disease-free survival, although this study was not specifically powered to answer this question [22]. Given the uncertainty regarding chemotherapy benefit in low-clinical-risk patients and our observation of the low probability of obtaining a recurrence score ≥ 26 in this group, we would suggest that restricting clinical use of molecular assays to patients with high clinical risk would be a rational approach.

There are limitations to our study. In terms of design, any study that is poised to influence physician decision-making processes could have considered an alternative design at a time when an Oncotype DX was only funded for lymph node-negative tumors. This could have included a simple randomized trial of PREDICT 2.1 plus or minus Oncotype DX. However, such as design would not have accounted for confounding due to physician standard practice as it would not be possible to prevent physicians who always use PREDICT 2.1 (or Oncotype DX) from doing so. Alternatively, a cluster randomized trial could be performed; however, the number of different clusters (i.e., treatment centers) required for such a trial was not realistically feasible. As approximately half of the physicians were already using PREDICT 2.1 prior to the educational intervention, the introduction of this study tool may have had a lower-than-anticipated impact. Another limitation may be that the proportion of premenopausal patients in our cohort is low at 13%. However, as the study included all patients with newly diagnosed early-stage, lymph node-negative, breast cancer who are eligible for Oncotype DX testing under the established funding criteria in Ontario, Canada, there was no exclusion based on menopausal status. Interestingly, unlike the TAILORx, our study separates pre- and perimenopausal patients (these are pooled together in TAILORx [18]. As 13% of our cohort were premenopausal and 7% perimenopausal, the total of 20% is close to that reported in TAILORx. Finally, in our PREDICT 2.1 analysis, one could argue that we could have selected 3rd generation chemotherapy over 2nd generation. We elected against this as 2nd generation encompasses anthracycline-sparing regimens (e.g., docetaxel–cyclophosphamide), which would be much more likely to be selected as systemic therapy in this population of patients. Similarly, our current study did not incorporate the role of ki67 as a potential tool available at some centers that may have both prognostic and predictive roles in the management of breast cancer [23].

## 5. Conclusions

In this prospective cohort study, we found that providing PREDICT 2.1 results and an educational intervention did not alter the ordering of an Oncotype DX. However, we were able to provide evidence that low-risk patients, either through clinico-pathologic criteria or PREDICT 2.1, are less likely to meaningfully benefit from ordering an Oncotype DX. Restricting molecular assay use to higher-risk patients may be a reasonable approach to better utilize health-care resources.

## Figures and Tables

**Table 1:** Patient demographics with clinical and pathological features for all patients and through months 0–3, 4–6, 7–9, and 10–12 in different stages of the study.

		All Pts	Months 0–3	Months 4–6	Months 7–9	Months 10–12
N	All	602	198	123	125	156
Age (years)	Mean (sd)	63.2 (11.8)	62.3 (12.6)	64.5 (11.7)	64.6 (11.2)	62.3 (11.2)
	Median (IQR)	64 (55–71)	64 (53–71)	65 (57–72)	65 (57–72)	63 (54–70)
Tumor Size (mm)	Median (range)	16.5 (6–109)	17 (6–109)	17 (6–96)	15 (6–76)	17 (6–46)
Diagnosis to Resection months	Median (range)	1.2 (0, 4.7)	1.2 (0.2, 3.7)	1.0 (0.1, 4.7)	1.1 (0, 3.4)	1.3 (0.1, 4.5)
Sex	N (%) Female	596 (99.0)	197 (99.5)	122 (99.2)	124 (99.2)	153 (98.1)
Menopausal Status (of females)	N (%) Pre	80 (13.4)	33 (16.8)	15 (12.3)	11 (8.9)	21 (13.7)
	Peri	42 (7.0)	20 (10.1)	7 (5.7)	5 (4.0)	10 (6.4)
	Post-	474 (79.5)	144 (73.1)	100 (82.0)	108 (87.1)	122 (79.7)
Tumor Detection	N (%) Screening	394 (65.4)	125 (63.1)	74 (60.2)	93 (74.4)	102 (65.4)
	Symptomatic	208 (34.6)	73 (36.9)	49 (39.8)	32 (25.6)	54 (34.6)
ER Staining	N (%) None	0	0	0	0	0
	Weak	9 (1.5)	5 (2.5)	2 (1.6)	0	2 (1.3)
	Moderate	58 (9.6)	16 (8.1)	18 (14.6)	11 (8.8)	13 (8.3)
	Strong	535 (88.9)	177 (89.4)	103 (83.7)	114 (91.2)	141 (90.4)
PR Status	N (%) Positive	532 (88.4)	178 (89.9)	110 (89.4)	108 (86.4)	136 (87.2)
	Negative	70 (11.6)	20 (10.1)	13 (10.6)	17 (13.6)	20 (12.8)
PR Staining	N (%) None	70 (11.6)	20 (10.1)	13 (10.6)	17 (13.6)	20 (12.8)
	Weak	10 (1.7)	3 (1.5)	1 (0.8)	3 (2.4)	3 (1.9)
	Moderate	105 (17.4)	38 (19.2)	20 (16.3)	17 (13.6)	30 (19.2)
	Strong	417 (69.3)	137 (69.2)	89 (72.4)	88 (70.4)	103 (66.0)
Grade	N (%) 1	114 (18.9)	49 (24.8)	21 (17.1)	19 (15.2)	25 (16.0)
	2	370 (61.5)	109 (55.1)	76 (61.8)	87 (69.6)	98 (62.8)
	3	118 (19.6)	40 (20.2)	26 (21.1)	19 (15.2)	33 (21.2)
Histology	N (%) Ductal and NOS	445 (73.9)	143 (72.2)	87 (70.7)	92 (73.6)	123 (78.9)
	Classic lobular	69 (11.5)	19 (9.6)	20 (16.3)	15 (12.0)	15 (9.6)
	Mixed ductal–lobular	38 (6.3)	21 (10.6)	9 (7.3)	4 (3.2)	4 (2.6)
	Pleomorphic lobular	2 (0.3)	1 (0.5)	0	0	1 (0.6)
	Tubular	3 (0.5)	1 (0.5)	1 (0.8)	1 (0.8)	0
	Papillary	11 (1.8)	3 (1.5)	0	6 (4.8)	2 (1.3)
	Other	34 (5.7)	10 (5.1)	6 (4.9)	7 (5.6)	11 (7.1)
Isolated Tumor Cells	N (%) Yes	28 (4.7)	9 (4.6)	8 (6.5)	6 (4.8)	5 (3.2)
	No	494 (82.1)	167 (84.3)	97 (78.9)	102 (81.6)	128 (82.1)
	Unknown	80 (13.3)	22 (11.1)	18 (14.6)	17 (13.6)	23 (14.7)
Micrometastatic Disease	N (%) Yes	25 (4.2)	9 (4.6)	2 (1.6)	4 (3.2)	10 (6.4)
	No	571 (94.9)	185 (93.4)	120 (97.6)	120 (96.0)	146 (93.6)
	Unknown	6 (1.0)	4 (2.0)	1 (0.8)	1 (0.8)	0
Lymphovascular Invasion Present	N (%) Yes	69 (11.5)	23 (11.6)	17 (13.8)	14 (11.2)	15 (9.6)
	No	495 (82.2)	164 (82.8)	101 (82.1)	105 (84.0)	125 (80.1)
	Unknown	38 (6.3)	11 (5.6)	5 (4.1)	6 (4.8)	16 (10.3)

sd = standard deviation, IQR = interquartile range.

**Table 2:** PREDICT 2.1 results through months 0–3, 4–6, 7–9, and 10–12 in different stages of the study.

		Months 0–3	Months 4–6	Months 7–9	Months 10–12
N		198	123	125	156
Surgery Only	10-year OS%	74.7 (16.1)	72.6 (17.0)	73.9 (16.0)	76.1 (15.0)
Chemotherapy (2nd Generation)	Additional OS Benefit	1.24 (0.91)	1.23 (0.83)	1.20 (0.94)	1.23 (0.85)
	10-year OS%	78.5 (15.9)	76.5 (16.9)	77.3 (16.0)	79.8 (14.7)
Chemotherapy (3rd Generation)	Additional OS Benefit	2.06 (1.52)	2.03 (1.38)	1.90 (1.27)	2.02 (1.42)
	10-year OS%	79.2 (15.9)	77.2 (16.8)	78.0 (16.0)	80.6 (14.7)
Hormone Therapy	Additional OS Benefit	2.56 (1.79)	2.53 (1.65)	2.38 (1.51)	2.53 (1.69)
	10-year OS%	77.3 (15.9)	75.2 (16.8)	76.1 (16.0)	78.6 (14.7)
Bisphosphonates	Additional OS Benefit	0.78 (0.57)	0.77 (0.54)	0.74 (0.52)	0.72 (0.49)
	10-year OS%	76.6 (16.1)	75.3 (17.0)	76.6 (16.1)	79.0 (15.2)
No Cancer	10-year OS%	83.1 (16.3)	81.0 (17.3)	81.5 (16.4)	84.4 (14.9)
PREDICT 2.1 10-year Chemo 2nd gen OS Survival Benefit	0–1%	67 (54.5)	70 (56.0)	92 (59.0)	343 (57.0)
	>1 to 2%	39 (31.7)	32 (25.6)	42 (26.9)	166 (27.6)
	>2 to 3%	10 (8.1)	5 (4.0)	13 (8.3)	47 (7.8)
	>3%	7 (5.7)	7 (5.6)	9 (5.8)	35 (5.8)
Clinical Risk	N (%) High	44 (35.8)	50 (40.0)	56 (35.9)	223 (37.0)
	Low †	79 (64.2)	75 (60.0)	100 (64.1)	379 (63.0)

OS = overall survival. † (size ≤ 30 and grade 1 OR, size ≤ 20 and grade 2 OR, and size ≤ 10 and grade 3).

**Table 3:** Oncotype DX^®^ recurrence score, clinical risk, Predict 2.1 overall survival benefit from 2nd generation chemotherapy, and adjuvant treatment for patients through months 0–3, 4–6, 7–9, and 10–12 in different stages of the study.

		Months 0–3	Months 4–6	Months 7–9	Months 10–12	*p* Value *
N		198	123	125	156	
N (%) of patients who had an Oncotype DX^®^ Ordered		92 (46.5)	62 (50.4)	50 (40.0)	74 (47.4)	0.37
Oncotype DX^®^ Recurrence Score	Mean (sd)	18.7 (12.4)	15.6 (8.6)	17.6 (9.1)	19.2 (12.0)	0.25
	Median (IQR)	15 (11, 22)	13.5 (9, 20)	16 (12, 22)	16 (12, 23)	
Oncotype DX^®^ Recurrence Score	N (%) high risk (≥26)	19 (20.7)	8 (12.9)	10 (20.0)	16 (21.6)	0.54 #
	Intermediate risk (21 to 25), and	10 (10.9)	5 (8.1)	7 (14.0)	8 (10.8)	
	Low risk (≤20)	63 (68.5)	49 (79.0)	33 (66.0)	50 (67.6)	
Resection to Treatment	Median (RANGE) Months	4.3 (0.1, 12.2)	3.4 (0.5, 9.7)	3.2 (0.5, 11.7)	2.7 (0.4, 9.5)	0.004
Chemotherapy Planned	N (%) Yes	25 (12.6)	11 (8.9)	13 (10.4)	28 (17.9)	0.22 #
	No	172 (86.9)	105 (85.4)	99 (79.2)	112 (71.8)	
	Unknown	1 (0.5)	7 (5.7)	13 (10.4)	16 (10.3)	
Recommended Chemotherapy and Frequency	2nd Generation	17 (68.0)	10 (83.3)	16 (94.1)	20 (74.1)	0.42
	3rd Generation	8 (32.0)	2 (16.7)	1 (5.9)	7 (25.9)	
Recommended Chemotherapy and Frequency	TC	17 (68.0)	10 (83.3)	15 (88.2)	20 (74.1)	0.60 #
	AC	0	0	1 (5.9)	0	
	Dd AC-paclitaxel	3 (12.0)	1 (8.3)	0	4 (14.8)	
	Dd AC-weekly paclitaxel	2 (8.0)	1 (8.3)	1 (5.9)	1 (3.7)	
	AC-weekly paclitaxel	0	0	0	1 (3.7)	
	AC-docetaxel	2 (8.0)	0	0	0	
	FEC-D	1 (4.0)	0	0	1 (3.7)	
Chemotherapy Received	N (%) Yes	22 (11.1)	10 (8.1)	14 (11.2)	23 (14.7)	0.25
Resection to Chemotherapy	Median (RANGE) Months	1.9 (1.0, 3.0)	1.8 (0.2, 2.5)	1.8 (1.3, 2.4)	1.9 (0.7, 3.2)	0.86
Radiation Received	N (%) Yes	112 (56.6)	88 (71.5)	90 (72.0)	110 (70.5)	0.025
Resection to Radiation	Median (RANGE) Months	2.4 (1.0, 7.9)	2.2 (1.0, 8.1)	2.1 (1.0, 6.2)	2.3 (0.9, 9.1)	0.72
Endocrine Therapy Received	N (%) Yes	169 (85.4)	104 (84.6)	110 (88.0)	130 (83.3)	0.91
Resection to Endocrine Therapy	Median (RANGE) Months	1.8 (0.0, 8.2)	1.9 (0.5, 7.1)	2.3 (0.6, 7.6)	2.2 (0.5, 9.8)	0.04
Clinical Risk	N (%) High	46 (50.0)	32 (51.6)	25 (50.0)	35 (47.3)	0.72
	Low †	46 (50.0)	30 (48.4)	25 (50.0)	39 (52.7)	
Predicted 10-Year Survival	N (%) ≥ 92%	52/92 (56.5)	29/62 (46.8)	17/45 (37.8)	38/74 (51.4)	0.08
PREDICT 2.1 10-year Chemo 2nd gen OS Survival Benefit	0–1%	35 (38.0)	19 (30.7)	19 (42.2)	31 (41.9)	0.26 #
	>1 to 2%	36 (39.1)	28 (45.2)	20 (44.4)	30 (40.5)	
	>2 to 3%	12 (13.0)	9 (14.5)	3 (6.7)	8 (10.8)	
	>3 to 5%	9 (9.8)	6 (9.7)	3 (6.7)	5 (6.8)	
	>5%	0 (0)	0 (0)	0 (0)	0 (0)	

sd = standard deviation, IQR = interquartile range, TC = docetaxel/cyclophosphamide, AC = doxorubicin/cyclophosphamide, FEC-D = 5FU/epirubicin/cyclophosphamide/docetaxel, OS = overall survival. † (size ≤ 30 and grade 1 OR size ≤ 20 and grade 2 OR size ≤ 10 and grade 3). * *p*-values based on comparing 0–6 months versus 7–12 months. # categories were dichotomized for statistical power purposes; tests compared high risk vs. intermediate/low risk, chemotherapy planned vs. no/unknown, TC vs. other, and 0–1% vs. >1%.

**Table 4:** Physician questionnaire before and after educational intervention.

		Months 0–3	Months 4–6	Months 7–9	Months 10–12
N		198	123	125	156
N with Questionnaires		0	123	0	156
Was Oncotype DX Recurrence Score Available When You Saw Patient?	N (%) Yes	NA	7 (5.7)	NA	6 (3.9)
Did you use PREDICT2.1 tool results?	N (%) Yes	NA	64 (52.5)	NA	135 (86.5)
Did you order Oncotype DX?	N (%) Yes	NA	60 (49.6)	NA	71 (45.8)
Did you recommend chemotherapy?	N (%) Yes	NA	6 (4.9)	NA	12 (7.7)
	No		68 (55.3)		82 (52.6)
	Pending Oncotype		49 (39.8)		62 (39.7)
Reason for No Chemotherapy	No benefit based on clin/path	NA	53 (77.9)	NA	48 (60.0)
	No benefit based on PREDICT		8 (11.8)		14 (17.5)
	Patient preference		6 (8.8)		10 (12.5)
	Patient comorbidities		1 (1.5)		8 (10.0)
Unsure whether adjuvant chemotherapy would be best	Strongly Disagree	NA	30 (24.6)	NA	39 (25.3)
	Disagree		39 (32.0)		35 (22.7)
	Neither Agree nor Disagree		11 (9.0)		16 (10.4)
	Agree		37 (30.3)		58 (37.7)
	Strongly Agree		5 (4.1)		6 (3.9)
PREDICT results make me more confident in my recommendation	Strongly Disagree	NA	1 (1.4)	NA	3 (2.0)
	Disagree		8 (11.0)		12 (7.8)
	Neither Agree nor Disagree		21 (28.8)		36 (23.5)
	Agree		31 (42.5)		71 (46.4)
	Strongly Agree		12 (16.4)		31 (20.3)
PREDICT tool provided additional clinically relevant information	Strongly Disagree	NA	0	NA	3 (2.0)
	Disagree		8 (11.0)		8 (5.2)
	Neither Agree nor Disagree		10 (13.7)		32 (20.9)
	Agree		45 (61.6)		85 (55.6)
	Strongly Agree		10 (13.7)		25 (16.3)
PREDICT influenced my treatment recommendation	Strongly Disagree	NA	3 (4.2)	NA	7 (4.6)
	Disagree		11 (15.3)		21 (13.7)
	Neither Agree nor Disagree		17 (23.6)		47 (30.7)
	Agree		32 (44.4)		56 (36.6)
	Strongly Agree		9 (12.5)		22 (14.4)
I would use PREDICT tool again	Strongly Disagree	NA	1 (1.4)	NA	1 (0.7)
	Disagree		1 (1.4)		5 (3.3)
	Neither Agree nor Disagree		3 (4.1)		19 (12.4)
	Agree		41 (56.2)		68 (44.4)
	Strongly Agree		27 (37.0)		60 (39.2)

## Data Availability

All data are provided in the tables and Supplemental Materials. Requests for datasets can be made to the Principal Investigator with permission from the Ontario Cancer Research Ethics Board (OCREB).

## Supplementary Materials

The following supporting information can be downloaded at: https://www.mdpi.com/article/10.3390/curroncol31030096/s1, S1: Protocol; S2: Physician Questionnaire; S3: Physician Intervention.
