# Supplementary material for: Does Pre-Emptive Availability of PREDICT 2.1 Results Change Ordering Practices for Oncotype DX? A Multi-Center Prospective Cohort Study
Source: Curr Oncol. 2024 Feb 27;31(3):1278–90. doi: 10.3390/curroncol31030096 (PMC10969492; doi:10.3390/curroncol31030096)
Supplement: Supplementary file 1 [file curroncol-31-00096-s001.zip › Appendix 1 REaCT-Algorithm Protocol 14 Jan 2020.pdf]

14 January 2020

A multi-centre, prospective, observational study evaluating whether integration of prognostic and predictive algorithms into routine clinical practice effect whether oncologists order multigene assays in patients with early stage breast cancer. (REaCT-Algorithm)

Sponsor: The Ottawa Hospital Research Institute
725 Parkdale Avenue
Ottawa, ON, K1Y 4E9
Coordinating Centre: The Ottawa Hospital Cancer Centre

Qualified Co-Investigators:

Dr. Mark Clemons MD, FRCPC (Medical Oncology)
Division of Medical Oncology, Dept of Medicine
The Ottawa Hospital Cancer Centre
501 Smyth Road
Ottawa, Ontario K1H 8L6
Tel: 613-773-7700 ext 70170
Fax: (613) 247-3511
E-mail: mclemons@toh.ca

Dr. Arif Ali Awan, MDCM, FRCPC (Medical Oncology)
Division of Medical Oncology, Dept of Medicine
The Ottawa Hospital Cancer Centre
501 Smyth Road, C2416
Ottawa, Ontario K1H 8L6
Tel: 613-798-5555 x. 70181
Fax: (613) 247-3511
E-mail: aawan@toh.ca

Co-investigators:
John Hilton
Demetrios Simos
Mohammed FK Ibrahim
Caroline Hamm
Nadia Califaretti
Andrew Robinson
Angel Arnaout
Susan Robertson
Brian Hutton
Dean Fergusson,
Greg Pond

Kednapa Thavorn

Funding source: OICR Health Services Research Network grant P.HSR.159

Clinical Trial Registration: NCT 04131933

Protocol Date: 14 January 2020

PROTOCOL SIGNATURE PAGE

My signature below confirms that I have reviewed and approved this protocol and agree that it contains all necessary details for carrying out the study as described. I will conduct this protocol as outlined therein, and according to Good Clinical Practice and all applicable local regulations

____
Qualified Investigator (Please Print)

____
Qualified Investigator Signature

____
Date

Table of Contents

1.0 SUMMARY 5

2.0 INTRODUCTION 6

3.0 METHODS 12

3.1 Study Design 12

3.2 Study Population and Eligibility 12

3.3 Study Procedures 13

3.4 Study Schedule 15

3.5 Physician Participation 19

3.6 Study Objectives 19

3.7 Study Outcomes 20

3.8 Data Collection 21

4.0 STATISTICAL ANALYSIS 22

5.0 SAMPLE SIZE 24

6.0 STUDY TIMELINE 25

7.0 REFERENCES 27

1.0 SUMMARY

A broad range of prognostic and predictive tools are available for patients with newly diagnosed early stage hormone receptor positive, Her2 negative breast cancer. These range from free and publicly available mathematical algorithms (e.g. NHS Predict, Magee formulae, Gage and Tennessee equations) that incorporate standard pathology results, through to expensive genomic tests (e.g. Oncotype DX ® and Endopredict ®). It is not known how the use of these different scores affects physician decision making with respect to ordering genomic tests, nor how well these algorithms predict for the results of Oncotype DX ® in the real-world setting. This pragmatic study will help to answer these questions.

2.0 INTRODUCTION

A broad range of prognostic and predictive tools have been developed for patients with newly diagnosed early stage breast cancer [1-4]. These include free and publicly available algorithms (e.g. NHS Predict 2.1 [5], Adjuvant online! [6-8], the Magee equations (1,2,3) [9, 10], Gage [11] and University of Tennessee probability models [12]), IHC4 [13] that incorporate standard readily available pathology results to genomic tests such as Oncotype DX ®, MammaPrint ®, EndoPredict ®, Breast Cancer Index ®.

Oncotype DX ®

In recent years there has been widespread introduction of multigene genomic assays and immunohistochemistry (IHC)-based scores to improve clinical decision making for patients with early stage hormone receptor positive, Her2 negative breast cancer [14-18]. The Oncotype DX ® Recurrence Score (ODX; Genomic Health, Redwood City, CA) is a widely used prognostic and predictive tool to guide treatment in patients with node-negative, estrogen receptor (ER) positive and/or progesterone receptor (PR) positive disease. Among genomic tests, Oncotype DX ® and MammaPrint ® are prospectively validated at present time [19, 20]. While Oncotype DX ® use has been shown to reduce the overall use of adjuvant chemotherapy, its use presents challenges which include: its expense (C$4380) restricting its use to resource rich countries significantly affects its potential for use in developing countries, in addition, as the test requires a tissue specimen to be sent to a central laboratory thereby increasing

local pathology service demands, it leads to a delay in making treatment decisions. Oncotype Dx use also results in higher odds of chemotherapy use in patients with small node-negative cancers, lower odds of use of chemotherapy in node-positive or large node-negative disease, and an increase in the proportion of test requests in the traditional intermediate risk group (i.e. Oncotype DX ® Recurrence Score (RS) of 18-30) the group for which until recently there were no definitive answers with respect to the benefits of chemotherapy [16, 20].

While the results of the TAILORx trial showed that overall patients in the intermediate risk group derived no additional benefit from chemotherapy, the subsequent subgroup analyses have added further confusion for patients and health care providers [20]. In this trial, patients in the intermediate risk group (adjusted to 11-25 for the trial design) were randomised to endocrine therapy with or without chemotherapy. This trial included 9719 patients aged 18-75 with 1.1-5 cm primary tumours, or 0.4-1.00 cm tumors with unfavourable features. Of the randomised patients 62% had 1.1-2 cm cancers, 80% had low-intermediate grade tumours and 68% were considered to have clinically low risk disease [6-8] The results showed that postmenopausal (defined as those >50 years of age) derived no benefit from adding adjuvant chemotherapy to endocrine therapy with Oncotype DX ® RS of 11-25. While in patients aged 50 and under there appeared to be some benefit for chemotherapy if they had “high”-intermediate Oncotype DX ® RS of 21-25. The final recommendations were that for patients older than 50 years with Oncotype DX ® RS of 0-25, there is no benefit of

adjuvant chemotherapy,while for those aged 50 years or younger Oncotype DX ® RS of 21-25 there was a 6.3% benefit in invasive disease-free survival at 9 years from addition of adjuvant chemotherapy. Furthermore, Oncotype DX ® does not routinely incorporate classic histo-pathological data and could predict the same benefit from chemotherapy for a patient with 0.6 cm and 5.0 cm breast cancer while the benefit of chemotherapy is substantially more for a patient 5.0 cm breast cancer. This is reflected in a recent analysis of the TAILORX data which incorporates clinical risk demonstrating that clinical risk is prognostic and patients with lower clinical risk had a lower magnitude of benefit from treatment [21].

While the TAILORx results have received significant media attention, we believe there are important unanswered questions and potential limitations concerning the widespread adoption of Oncotype DX ® . Many of these were raised by the study discussant at the 2018 ASCO meeting [22]. These included:

1. The study failed to evaluate which patients do not need the Oncotype DX ® test performed.
2. Decision points for patients with newly diagnosed breast cancer are performed at baseline (addressed by TAILORx) and the duration of endocrine therapy is not addressed [23].
3. As the study enrolled patients at low clinical risk of recurrence, we do not know the optimal management of patients with stage II-III disease.

In addition, there is considerable variation in the ordering of Oncotype between physicians and between cancer centres in Ontario. A recent survey showed that some centres order Oncotype Dx in over 90% of patients, while others order it in around 15% of eligible patients. As use of this test costs around $9,000,000 a year in Ontario alone (and likely to rise with the recent TAILORx results) this equipoise has considerable impact on both patient care and financial impact on the health care system. **There needs to be a more concerted effect to improve both patient care, as well as the spiralling costs of this test, by prospectively identifying those patients most likely to benefit from the performance of Oncotype DX ® testing**.

PREDICT 2.1

PREDICT breast cancer prognostication and treatment prediction model is an online tool available at https://breast.predict.nhs.uk/tool. It was originally developed in 2010 using data from the patients in the United Kingdom and then validated from independent case series in British Columbia, Malaysia, Netherlands and two additional cohorts from United Kingdom [24] consisting of 11,272 patients. It uses readily available features of patients with breast cancer such as age, menopause status, tumor size, lymph node involvement, grade, estrogen receptor status, Her2 status, Ki67 (if available), tumor detection method to instantly predict patient's overall survival at 5, 10 at 15 years to provide prognosis. In additional, it uses the results of high-quality meta-analysis from Early Breast Cancer Trialists Collaborative Group to provide the estimated

overall survival benefit from different modalities of breast cancer treatment such as endocrine therapy and second/third generation adjuvant chemotherapy. It is the only breast cancer prognostic model currently available online and has been used over 1 million times. Although the recent version of the PREDICT model better predicts mortality for younger patients with breast cancer, it also has limitations. PREDICT was originally developed based on data from patients between 1999-2003 and since then multiple advances such as the use of trastuzumab and third generation adjuvant chemotherapy regimens which include taxane have improved outcomes. The model has since been updated to reflect these advances. In addition, PREDICT uses classic histo-pathological features to determine prognosis and data from meta-analysis to determine benefit based on population outcomes rather than genomic assays which assess individual genomic alterations in a patient's tumor. Furthermore, both these models do not incorporate patient's co-morbidities especially in an aging population with other chronic diseases.

In the current prospective trial, we will evaluate whether use of PREDICT 2.1impacts physician decision making with respect to ordering Oncotype DX ®, particularly in patients aged <50, and other important cancer care metrics including chemotherapy use, time to commencement of chemotherapy, and resource utilisation. This data will also be used to provide further validation of the algorithms in a real-world, multi-centre setting.

Alternative Trial Designs

Any study that is poised to influence physician decision making processes could have multiple alternative designs. The study group has experience in performing trials evaluating the use of different risk models in subsequent Oncotype DX ® ordering [25]. Designs could include a simple randomised trial of Predict plus or minus Oncotype DX ®. However, this would not account for confounding due to physician standard practice as it would not be possible to prevent physicians who always use PREDICT 2.1 (or Oncotype DX ®) from doing so. Alternatively, a cluster randomized trial could be performed, however, the number of different clusters (i.e. treatment centres) required for such a trial make it prohibitive. Simply put, this is a complicated question to address and any design will not be perfect. However, we feel the current proposal has the greatest chance to broadly impact on patient care, physician practice and health care economics.

Ethical Considerations

The ethics of this type of study have been considered at some length by the study team and the methodologists. The previously published Magee study (31) had a very similar study design and evaluated the effect of another mathematic model on physician decision making regarding Oncotype DX ® ordering. Requesting consent from either patients or physicians will add inherent bias to the study as the findings will then not reflect broader clinical practice. As both

PREDICT 2.1 and Oncotype DX ® are available for all eligible patients this bias could significantly impact the results and must be avoided.

3.0 METHODS

3.1 Study Design

A multi-center prospective cohort study, with a pre- versus post-comparison, evaluating how making PREDICT 2.1 results available to treating physicians impacts on subsequent ordering of Oncotype DX ®, chemotherapy use, time to commencement of chemotherapy, and health care resource utilization. In addition, in order to understand physician reasoning and comfort with making systemic therapy decisions with either PREDICT 2.1, Oncotype DX ® or both, a physician questionnaire will be performed in order to drive subsequent knowledge mobilisation strategies.

The study will involve participation from 6-8 Ontario sites that will be chosen to reflect a diverse patient population in Ontario and the site’s involvement in the REaCT program.

3.2 Study Population and Eligibility

Patients that are eligible for Oncotype DX ® testing under current funding parameters with complete pathology reports for newly diagnosed early stage breast cancer will be eligible for this prospective trial. Patients that are ineligible for OncoType DX ® testing but insist on testing and choose to self-pay, are not eligible for this study.

Inclusion criteria:

- histologically confirmed primary breast cancer
- no prior chemotherapy
- eligible for Oncotype DX ® testing as per current Ontario funding criteria including: ER positive, PR positive or negative, HER2 negative, lymph node status negative or micro-invasive disease, tumour >1 cm in size (or if ≤1cm, must be grade 2/3 or have lymph node micrometastasis).

Exclusion criteria:

- neoadjuvant treatment including window of opportunity trials
- recurrent breast cancer

3.3 Study Procedures

Each site will have a different process for determining eligibility. Research staff at each site will be confirming Oncotype DX ® eligibility from pathology reports and will be working with New Patient booking staff (or similar) to get weekly/daily clinic lists of new patients whose tumors meet eligibly for Oncotype DX ® testing.

Patient tumours at each site will enter study screening when a complete pathology report is received. Once a patient tumour is deemed eligible, it is given a Study ID number. Pathology data is collected by study staff. At all points of the study, patients with eligible tumours will meet with their treating physician to discuss the individualised testing and treatment plan that is best for them.

Once the physician meets with the patient, we will also collect the chemotherapy treatment plan details.

After 3 months of activation, physicians will start completing a questionnaire for each new patient with an eligible tumour. Delaying the questionnaires for the first 3 months is to prevent bias.

After 6 months of activation, the physicians will receive a teaching session (Intervention) consisting of PowerPoint slides which will present an updated review on the benefits and limitations of Oncotype DX and PREDICT testing.

After this teaching session, study staff at each site will be using the pathology data to run the PREDICT tool for each patient and will provide the results to each physician prior to his/her meeting with each patient. Again after 3 months, we will re-introduce the physician questionnaire for the last 3 months of the study.

In summary:

Month 1 to 3: pathology and chemo data collected, NO physician questionnaire

Month 4 to 6: pathology and chemo data collected, PLUS physician questionnaire

Intervention teaching after 6 months of study activation

Month 7 to 9: pathology and chemo data collected, PREDICT 2.1 tool used, NO physician questionnaire

Month 10 to 12: pathology and chemo data collected, PREDICT 2.1 tool used, PLUS physician questionnaire

3.4 Study Schedule

<table>
<tr><th colspan="2">Study phase</th><th>Patient activities</th><th>Physician activities</th></tr>
<tr><td rowspan="2">Pre-intervention</td><td>Baseline to month 3</td><td rowspan="2">Data Collection: there's no direct patient involvement. The CRA will collect de-identified information from patient's health record regarding:
Eligibility for Oncotype DX ® (ODX) reimbursement by OHIP (histologically confirmed primary breast cancer, no prior chemotherapy, ER positive, PR positive or negative, HER2 negative, lymph node status negative or micro-invasive disease, tumour >1 cm in size (or if ≤1cm, must be grade 2/3 or have lymph node micrometastasis))
Whether ODX was ordered and if so, the ODX recurrence score
Data required for Predict (age, menopause, ER/Her2/Ki67 status, tumor size, tumor grade, method of detection, number of positive lymph nodes)
Which chemotherapy regimen was planned and what chemotherapy the patient received
Time from medical oncologist visit to starting chemotherapy</td><td></td></tr>
<tr><td>Month 4 to 6</td><td>Questionnaire and Data Collection: Starting at month 4, once a physician has decided on the systemic therapy recommendation for each patient, the physicians will be asked to complete a questionnaire to assess their comfort with this recommendation. The following will be recorded:
Whether ODX was ordered
Whether Predict 2.1 was used</td></tr>
<tr><td>Intervention</td><td>At 6 months</td><td>No patient intervention</td><td>Physicians will be provided with educational material regarding the strengths and weakness of OncoTypeDX testing and the Predict 2.1 tool</td></tr>
<tr><td>Post-intervention</td><td>Months 6 to 9</td><td>Data Collection: exactly as the pre-intervention phase with no direct patient intervention. The CRA will collect de-identified information from patient's health record regarding:</td><td>Predict 2.1: CRA will use the Predict 2.1 tool for all patients that are potentially eligible for Oncotype DX testing. This result will be provided to the treating medical oncologist in the clinic.</td></tr>
</table>

	Month 10 to 12	• Eligibility for Oncotype DX ® (ODX) reimbursement by OHIP (histologically confirmed primary breast cancer, no prior chemotherapy, ER positive, PR positive or negative, HER2 negative, lymph node status negative or micro-invasive disease, tumour >1 cm in size (or if ≤1cm, must be grade 2/3 or have lymph node micrometastasis))<br>• Whether ODX was ordered and if so, the ODX recurrence score<br>• Data required for Predict (age, menopause, ER/Her2/Ki67 status, tumor size, tumor grade, method of detection, number of positive lymph nodes)<br>• Which chemotherapy regimen was planned and what chemotherapy the patient received<br>• Time from medical oncologist visit to starting chemotherapy	**Predict 2.1:** CRA will use the Predict 2.1 tool for all patients that are potentially eligible for Oncotype DX testing. This result will be provided to the treating medical oncologist in the clinic.<br>**Questionnaire and Data Collection:** Starting at month 10, once a physician has decided on the systemic therapy recommendation for each patient, the physicians will be asked to complete a questionnaire to assess their comfort this recommendation. The following will be recorded:<br>• Whether ODX was ordered<br>• Whether Predict 2.1 was used

Pre-intervention:

Baseline to Month 6: To obtain information on baseline use and physician practice pattern across multiple cancer centres, for the first 6 months of the study, the study CRA will use the patients' electronic medical record (EMR) to collect de-identified data for each new patient who is eligible for Oncotype DX testing, and whether or not the treating physician used Oncotype DX ® or Predict 2.1. During this 6-month period, the CRA will NOT provide the oncologist with PREDICT 2.1 results. If the physician wishes to use the PREDICT 2.1 tool on their patients, they will be allowed to do so. The CRA will record chemotherapy

treatment information and the time between seeing the medical oncologist and starting chemotherapy.

Months 4 to 6: Once a physician has made a decision about a patient's treatment, the physician will complete a questionnaire [25] to assess their comfort with their systemic therapy recommendations for each patient. Collecting this information from physicians after 3 months of baseline behaviour removes potential influence of the questionnaire prompting physicians to consider PREDICT 2.1 in their standard decision-making.

Intervention Teaching Session (at 6 months): All medical oncologists involved in the study will receive a teaching session to remind physicians of both the strengths and weaknesses of Oncotype DX ® and PREDICT 2.1 tools.

Post-Intervention

Months 6 to 12: In the second 6 months of the study, the CRA will use the PREDICT 2.1 tool for all patients that are potentially eligible for Oncotype DX ® testing. The PREDICT model results will be attached to each patient chart and therefore available to the treatment oncologist.

Months 10 to 12: Once a physician has made a decision about a patient's treatment, the physician will complete the questionnaires again [25] to assess their comfort with the systemic therapy recommendations for each patient.

This study timeline requires that each site will have its own study start date and pre-intervention and post-intervention periods. Throughout the entire study timeline, the study CRA at each site will collect de-identified routine diagnostic history and pathology information from the patient’s electronic medical record and record it in the study database.

Rationale:

The Pre- and Post-Intervention periods have been chosen to practically allow sufficient time for individual centres to accrue patients and to allow sufficient data to be available to reflect any effects of the teaching session within the allocated study-funding window.

As the study makes no treatment recommendations based on the PREDICT 2.1 model and the oncologist can use the PREDICT 2.1 tool at any time if they so wish, all patients in this study are receiving a standard of care discussion and treatment decisions with no additional risk. The study will therefore not involve consenting patients or physicians as all treatment decisions will be made by each patient and physician as per clinical standard of care. The waiver of consent model has been used in the past in REaCT trials [25], with great success as it avoids the significant issue of physician bias. REB application will be through OCREB and the trial will be registered on clinicaltrials.gov.

3.5 Physician Participation

It is our hope that all medical oncologists that see patients with early stage breast cancer will participate so that the study captures broad clinical practice at all participating sites. There is no recruitment target for physician participation.

As described above, during months 4-6 and 10-12 of the study, physicians will receive ONE questionnaire for EACH patient that would meet the eligibility criteria for Oncotype DX ® testing. There are two versions of the physician's questionnaire (Pre-Intervention at 4-6 months and Post-Intervention at 10-12 months). This questionnaire consists of 8-9 questions and should take less than 5 minutes to complete. The questionnaire will be identified with the patient Study ID but will not record the physician's name. There are no risks to the physician bin completing the questionnaire. The study will not collect any data on individual physicians' practice patterns but instead on each centre's practice as a whole. Completion of the questionnaire constitutes implied consent. A physician can also refuse to complete a questionnaire for a specific patient.

3.6 Study Objectives

Primary Objective:

To assess whether providing individual patient prognostic and predictive scores from PREDICT 2.1 affects the rate of subsequent requests for Oncotype DX testing.

Secondary Objectives:

To assess whether routine availability of PREDICT 2.1 affects: the frequency of chemotherapy use, type of chemotherapy use, the time to starting chemotherapy, endocrine therapy or radiation therapy and Oncotype DX ® cost and total health system costs and subsequent health care utilization. In order to use these study findings for knowledge mobilisation strategies, we need to understand physician's reasoning and comfort with making systemic therapy decisions. Physicians will therefore complete a questionnaire [25] after seeing each patient.

3.7 Study Outcomes

Primary Endpoint: The primary outcome will be the proportion of patients for which Oncotype DX ® is ordered, defined as the number of patients with Oncotype DX ® orders divided by the number of patients eligible for Oncotype DX ® testing.

Secondary Endpoints:

Frequency and type of chemotherapy used will be collected from the patient's electronic medical record as will timing to start chemotherapy, radiation therapy and endocrine therapy. Prognostic risk scores, including Magee formulae, Gage and Tennessee equations will be calculated using patient and tumour characteristics. These scores will also be compared with Oncotype DX ® results when performed. Physician questionnaire results will be used to assess physician comfort when making systemic therapy decisions.

Economic analysis:

We will conduct a cost analysis and compare total health system costs before and after the model implementation from the Ontario government's perspective. The cost components will include Oncotype DX cost (C$4380), adjuvant chemotherapy cost and other health services costs. We assum that the PREDICT 2.1 tool is readily available at no cost. The cost associated with other health services among patients who receive and those who do not receive adjuvant chemotherapy (including cost of cancer clinic, in-patient services, physician billing, chemotherapy, endocrine therapy, supportive drug, home care, in-patient and emergency department costs) [26] will be obtained from a concurrent cohort study and Canadian sources. Based on this analysis, we will then extrapolate the cost findings to the entire population of Ontario to report the financial implications of our intervention to Cancer Care Ontario.

3.8 Data Collection

Data collection begins when a pathology report is completed and deemed eligible for Oncotype testing under the Ontario criteria. The following information will be collected from the patient's electronic medical record:

- Eligibility for Oncotype DX ® (ODX) reimbursement by OHIP (histologically confirmed primary breast cancer, no prior chemotherapy, ER positive, PR positive or negative, HER2 negative, lymph node status negative or micro-invasive disease, tumour >1 cm in size (or if ≤1cm, must be grade 2/3 or have lymph node micrometastasis))

- Whether ODX was ordered and if so, the ODX recurrence score
- Data required for PREDICT 2.1 algorithm (age, menopause, ER/Her2/Ki67 status, tumor size, tumor grade, method of detection, number of positive lymph nodes)
- Which chemotherapy regimen was planned and what chemotherapy the patient received
- Time from medical oncologist visit to starting chemotherapy

Data collected will be de-identified (no patient identifiers) and will not be linked to the treating physician. Data will be entered by the CRA at each site into a secure study-specific database.

4.0 STATISTICAL ANALYSIS

The primary analysis will be based on a hierarchical logistic regression analysis, with time period (pre-intervention versus post-intervention) as the primary covariate of interest. For this model, patient characteristics are considered nested within treating physician, which is nested within treatment centre. Adjustments will be performed for baseline characteristics. From our survey, it was evident that there was significant variability between centres (and between physicians) for ordering Oncotype DX. Data from Ottawa shows that approximately 15% of approximately 600 patients treated by 8 physicians have this test performed. In contrast, Kingston (5 physicians) sees around 200 eligible patients a year and Oncotype DX is ordered for around 65% of these patients,

Thunder Bay (3 physicians) sees around 70 patients a year and also tests around 65% of eligible patients, and Newmarket (3 physicians) sees around 130 eligible patients a year and Oncotype DX is ordered for around 75% of these patients. Windsor (3 physicians) and Kitchener (4 physicians) both see around 150 patients per year and orders Oncotype DX testing on 90% of eligible patients. Given concerns that use of hierarchical clusters may not sufficiently account for the substantial variability between sites, analyses will also be conducted by site, grouped by baseline rate of testing (i.e. Kingston, Thunder Bay and Newmarket will be combined, as will Windsor and Kitchener; Ottawa will be a separate group on its own). A similar analysis will be performed using hierarchical logistic regression. In addition, key supportive analyses will also be performed using a two-sample chi-square test within each site separately, comparing the change in proportion of patients for which Oncotype DX is ordered for each physician (i.e. post-intervention proportion – pre-intervention proportion for physician A, B, C, …).

Analyses of secondary endpoints will be performed globally across all sites combined, and for each site individually. The chi-square test or Wilcoxon rank sum test will be used as appropriate for categorical or continuous endpoints respectively. Results will be presented using tables and figures, and exact confidence intervals will be constructed for outcomes of interest. Bootstrapping may be used for estimation purposes if required. All tests will be two-sided and statistical significance will be defined at the $\alpha=0.05$ level.

No interpolation will occur for missing data except in situations where the missing data is obvious (i.e. if a patient has missing Her2 status but receives trastuzumab, it can be assumed with confidence that the patient must be Her2 positive). No statistical adjustments will be made for multiple tests performed, however, appropriate caution will be made in interpretation of results, particularly for the analysis of secondary endpoints.

5.0 SAMPLE SIZE

Sample size calculations for hierarchical regression models are difficult, as they require estimates of the number of clusters, the size of each cluster, the effect of intervention on each cluster separately and intra-cluster correlation. Without prior information, these estimates are unlikely to be accurate. Hence, statistical power and sample size calculations will be based on practical considerations.

It is hypothesized that if the use of PREDICT 2.1 decreases the odds of Oncotype DX ordering by approximately 50% or more, the usage of Predict 2.1 will be of clinical importance. It is known that approximately 300 patients are seen in a given six-month period in Ottawa, and approximately 15% of eligible patients undergo Oncotype DX testing. For the other centres, the values are 75 and 75% (Newmarket), 75 and 90% (Windsor), 75 and 65% (Kingston), 35 and 65% (Thunder Bay), and 75 and 90% (Grand River). Based on these estimates,

a simple, two-sided, α=0.05, logistic regression model would have 80% power to detect an odds ratio of 0.46 (from 15% to 7.46%) in Ottawa, an odds ratio of 0.38 (from 90% to 77.4%) in Grand River/Windsor, and an odds ratio of 0.53 (from 70% to 55.4%) in Newmarket/Kingston/Thunder Bay. Overall, if one conservatively estimates at least 500 patients will be eligible pre-intervention and 500 patients will be eligible post-intervention, a simple logistic regression analysis would have over 80% power (2-sided, α=0.05) to detect an odds ratio of 0.67, assuming that approximately 50% of patients undergo Oncotype DX testing in the pre-intervention period. Combined, these estimates give assurance that the sample size for this study is sufficient to capture clinically meaningful effects.

6.0 STUDY TIMELINE

Funding to begin: Sept 1, 2019

Application to OCREB Sept 2019

REB approval Nov 2019

Site initiation visits Dec 2019-Feb 2020

Completion of initial 6-months of study May 2020

Educational intervention June 2020

Completion of second 6-months of study December 2020

Data analysis Jan-Feb 2021

Funding term end date: March 31, 2021

Table 1. Characteristics of study algorithms

**Algorithm name**	**Required clinic-path data**	**Formula**	**Population – LN pos and neg?**	**What it tells you**
NHS Predict 2.1	Age<br>Screen detected or not<br>Tumour size<br>LN involvement<br>Grade<br>ER<br>Her2<br>Ki67*	On line	Node pos<br>Node neg	10 yr distance disease free survival
Magee 1	Tumour size<br>Grade,<br>ER<br>PR<br>Her2<br>Ki-67	On line	Macro-node negative	**Surrogate Oncotype score**
Magee 2	Tumour size, grade, ER, PR, Her2 without Ki-67	On line	Macro-node negative	**Surrogate Oncotype score**
Magee 3	ER, PR, HER2<br>Ki-67	On line	Macro-node negative	**Surrogate Oncotype score**
Gage et al (2015)	ER<br>PR<br>Grade<br>simple algorithm based on tumor grade, PR >1% and ER>20%.			
University of Tennessee predictive algorithm	PR<br>Grade			**Surrogate Oncotype score**

7.0 REFERENCES

1. Pinder SE, Ellis IO, Elston CW: **Prognostic factors in primary breast carcinoma**. *Journal of clinical pathology* 1995, **48**(11):981-983.
2. Davies C, Godwin J, Gray R, Clarke M, Cutter D, Darby S, McGale P, Pan HC, Taylor C, Wang YC *et al*: **Relevance of breast cancer hormone receptors and other factors to the efficacy of adjuvant tamoxifen: patient-level meta-analysis of randomised trials**. *Lancet (London, England)* 2011, **378**(9793):771-784.
3. Goldhirsch A, Wood WC, Gelber RD, Coates AS, Thurlimann B, Senn HJ: **Progress and promise: highlights of the international expert consensus on the primary therapy of early breast cancer 2007**. *Annals of oncology : official journal of the European Society for Medical Oncology* 2007, **18**(7):1133-1144.
4. Galea MH, Blamey RW, Elston CE, Ellis IO: **The Nottingham Prognostic Index in primary breast cancer**. *Breast cancer research and treatment* 1992, **22**(3):207-219.
5. Mokbel K, Wazir U, El Hage Chehade H, Manson A, Choy C, Moye V, Mokbel K: **A Comparison of the Performance of EndoPredict Clinical and NHS PREDICT in 120 Patients Treated for ER-positive Breast Cancer**. *Anticancer research* 2017, **37**(12):6863-6869.
6. Campbell HE, Taylor MA, Harris AL, Gray AM: **An investigation into the performance of the Adjuvant! Online prognostic programme in early breast cancer for a cohort of patients in the United Kingdom**. *British journal of cancer* 2009, **101**(7):1074-1084.
7. Ravdin PM: **A computer based program to assist in adjuvant therapy decisions for individual breast cancer patients**. *Bulletin du cancer* 1995, **82 Suppl 5**:561s-564s.
8. Ravdin PM: **A computer program to assist in making breast cancer adjuvant therapy decisions**. *Seminars in oncology* 1996, **23**(1 Suppl 2):43-50.
9. Flanagan MB, Dabbs DJ, Brufsky AM, Beriwal S, Bhargava R: **Histopathologic variables predict Oncotype DX recurrence score**. *Modern pathology : an official journal of the United States and Canadian Academy of Pathology, Inc* 2008, **21**(10):1255-1261.
10. **Magee Equations™ for Estimating Oncotype DX® Recurrence Score** [http://path.upmc.edu/onlineTools/MageeEquations.html]
11. Gage MM, Rosman M, Mylander WC, Giblin E, Kim HS, Cope L, Umbricht C, Wolff AC, Tafra L: **A Validated Model for Identifying Patients Unlikely to Benefit From the 21-Gene Recurrence Score Assay**. *Clinical breast cancer* 2015, **15**(6):467-472.
12. Orucevic A, Bell JL, McNabb AP, Heidel RE: **Oncotype DX breast cancer recurrence score can be predicted with a novel nomogram using clinicopathologic data**. *Breast cancer research and treatment* 2017, **163**(1):51-61.
13. Cuzick J, Dowsett M, Pineda S, Wale C, Salter J, Quinn E, Zabaglo L, Mallon E, Green AR, Ellis IO *et al*: **Prognostic value of a combined estrogen receptor, progesterone receptor, Ki-67, and human epidermal growth factor receptor 2 immunohistochemical score and comparison with the Genomic Health recurrence score in early breast cancer**. *Journal of clinical oncology : official journal of the American Society of Clinical Oncology* 2011, **29**(32):4273-4278.
14. Sotiriou C, Pusztai L: **Gene-expression signatures in breast cancer**. *N Engl J Med* 2009, **360**(8):790-800.
15. Paik S, Shak S, Tang G, Kim C, Baker J, Cronin M, Baehner FL, Walker MG, Watson D, Park T *et al*: **A multigene assay to predict recurrence of tamoxifen-treated, node-negative breast cancer**. *N Engl J Med* 2004, **351**(27):2817-2826.
16. Paik S, Tang G, Shak S, Kim C, Baker J, Kim W, Cronin M, Baehner FL, Watson D, Bryant J *et al*: **Gene expression and benefit of chemotherapy in women with node-negative, estrogen receptor-positive breast cancer**. *Journal of clinical oncology : official journal of the American Society of Clinical Oncology* 2006, **24**(23):3726-3734.
17. Kelly CM, Bernard PS, Krishnamurthy S, Wang B, Ebbert MT, Bastien RR, Boucher KM, Young E, Iwamoto T, Pusztai L: **Agreement in risk prediction between the 21-gene recurrence score assay (Oncotype DX(R)) and the PAM50 breast cancer intrinsic Classifier in early-stage estrogen receptor-positive breast cancer**. *The oncologist* 2012, **17**(4):492-498.

18. Kelly CM, Krishnamurthy S, Bianchini G, Litton JK, Gonzalez-Angulo AM, Hortobagyi GN, Pusztai L: **Utility of oncotype DX risk estimates in clinically intermediate risk hormone receptor-positive, HER2-normal, grade II, lymph node-negative breast cancers**. *Cancer* 2010, **116**(22):5161-5167.
19. Cardoso F, van't Veer LJ, Bogaerts J, Slaets L, Viale G, Delaloge S, Pierga JY, Brain E, Causeret S, DeLorenzi M *et al*: **70-Gene Signature as an Aid to Treatment Decisions in Early-Stage Breast Cancer**. *N Engl J Med* 2016, **375**(8):717-729.
20. Sparano JA, Gray RJ, Makower DF, Pritchard KI, Albain KS, Hayes DF, Geyer CE, Jr., Dees EC, Goetz MP, Olson JA, Jr. *et al*: **Adjuvant Chemotherapy Guided by a 21-Gene Expression Assay in Breast Cancer**. *N Engl J Med* 2018.
21. Sparano JA, Gray RJ, Ravdin PM, Makower DF, Pritchard KI, Albain KS, Hayes DF, Geyer CE, Jr., Dees EC, Goetz MP *et al*: **Clinical and Genomic Risk to Guide the Use of Adjuvant Therapy for Breast Cancer**. *N Engl J Med* 2019, **380**(25):2395-2405.
22. **TAILORx: Endocrine Therapy Alone Is Sufficient for Most Patients With 'Intermediate-Risk' Breast Cancer** [http://www.ascopost.com/issues/june-10-2018/tailorx-endocrine-therapy-alone-in-intermediate-risk-breast-cancer/]
23. Sgroi DC, Sestak I, Cuzick J, Zhang Y, Schnabel CA, Schroeder B, Erlander MG, Dunbier A, Sidhu K, Lopez-Knowles E *et al*: **Prediction of late distant recurrence in patients with oestrogen-receptor-positive breast cancer: a prospective comparison of the breast-cancer index (BCI) assay, 21-gene recurrence score, and IHC4 in the TransATAC study population**. *The Lancet Oncology* 2013, **14**(11):1067-1076.
24. Candido Dos Reis FJ, Wishart GC, Dicks EM, Greenberg D, Rashbass J, Schmidt MK, van den Broek AJ, Ellis IO, Green A, Rakha E *et al*: **An updated PREDICT breast cancer prognostication and treatment benefit prediction model with independent validation**. *Breast cancer research : BCR* 2017, **19**(1):58.
25. Robertson SJ, Ibrahim MFK, Stober C, Hilton J, Kos Z, Mazzarello S, Ramsay T, Fergusson D, Vandermeer L, Mallick R *et al*: **Does integration of Magee equations into routine clinical practice affect whether oncologists order the Oncotype DX test? A prospective randomized trial**. *Journal of evaluation in clinical practice* 2019, **25**(2):196-204.
26. Mittmann N, Earle CC, Cheng SY, Julian JA, Rahman F, Seung SJ, Levine MN: **Population-Based Study to Determine the Health System Costs of Using the 21-Gene Assay**. *Journal of clinical oncology : official journal of the American Society of Clinical Oncology* 2018, **36**(3):238-243.
