# Supplementary material for: Does Pre-Emptive Availability of PREDICT 2.1 Results Change Ordering Practices for Oncotype DX? A Multi-Center Prospective Cohort Study
Source: Curr Oncol. 2024 Feb 27;31(3):1278–90. doi: 10.3390/curroncol31030096 (PMC10969492; doi:10.3390/curroncol31030096)
Supplement: Supplementary file 1 [file curroncol-31-00096-s001.zip › Appendix 2 REaCT-Algorithm Physician Questionnaire (Months 4 to 6) 14 Jan 2020.pdf]

A multi-centre, prospective, observational study evaluating whether integration of prognostic and predictive algorithms into routine clinical practice effect whether oncologists order multigene assays in patients with early stage breast cancer. REaCT-Algorithm

PHYSICIAN QUESTIONNAIRE (Months 4 to 6)

Note: Completion of this questionnaire constitutes implied consent.

Study ID: _______________

Date Questionnaire Completed (dd/mmm/yyyy): ________/________/________

Please take a moment to complete the following questionnaire regarding this patient and your current recommended adjuvant systemic therapy:

1. Was an Oncotype DX ® RecurrenceScore already available when you saw this patient? ☐No ☐Yes
2. Did you use the PREDICT 2.1 tool results? ☐No ☐Yes
3. Did you order Oncotype DX ®? ☐No ☐Yes
4. Did you recommend chemotherapy? ☐No ☐Yes ☐ Pending Oncotype Score

   If yes, chemo regimen recommended:
   ☐ 2nd generation →☐ TC ☐ AC
   ☐ 3rd generation →☐ dd AC-paclitaxel ☐ dd AC-weekly paclitaxel ☐ Other: _______
   ☐ FEC-D ☐ AC- weekly paclitaxel ☐ AC- docetaxel

   If no, please tick which apply:
   ☐ No clinically meaningful benefit from chemotherapy based on clinical-pathological features
   ☐ No clinically meaningful benefit from adjuvant chemotherapy based on PREDICT 2.1
   ☐ Patient preference for no chemotherapy
   ☐ Patient co-morbidities

Please indicate how strongly you agree or disagree with the following statements about your systemic treatment recommendations for this patient (select only one answer per question):

	Strongly Disagree	Disagree	Neither Disagree Nor Agree	Agree	Strongly Agree
5. I am unsure whether adjuvant chemotherapy would be best for this patient					
**If you used the PREDICT tool, please answer the following questions:**					
6. The PREDICT result makes me more confident in my treatment recommendation					
7. The PREDICT tool provided additional clinically relevant information					
8. The results of the PREDICT tool influenced my treatment recommendation					
9. I would use the PREDICT tool again					
