# Supplementary material for: Does Pre-Emptive Availability of PREDICT 2.1 Results Change Ordering Practices for Oncotype DX? A Multi-Center Prospective Cohort Study
Source: Curr Oncol. 2024 Feb 27;31(3):1278–90. doi: 10.3390/curroncol31030096 (PMC10969492; doi:10.3390/curroncol31030096)
Supplement: Supplementary file 1 [file curroncol-31-00096-s001.zip › Appendix 3 REaCT-Algorithm Physician Intervention 14 Jan 2020.pdf]

REACT ALGORITHM – EDUCATIONAL INTERVENTION

Co-PI: Drs. Mark Clemons and Arif Awan
The Ottawa Hospital
Version: 14 Jan 2020

SPARANO ET AL. "CLINICAL AND GENOMIC RISK TO GUIDE THE USE OF ADJUVANT THERAPY FOR BREAST CANCER" NEJM 2019: 380: 2395-2405

GENOMIC RISK AND CLINICAL RISK

Case 1: 60-year old patient with **5 cm**, node negative, grade 3, ER/PR positive, Her2 negative invasive ductal carcinoma

Case 2: 60-year old patient with **0.6 cm**, node negative, grade 3, ER/PR positive, Her2 negative invasive ductal carcinoma

Both these patients can have the same Oncotype DX ® Recurrence Score

Using PREDICT 2.1 (a mathematical tool that uses standard demographic and pathology results to calculate breast cancer prognosis and predict benefit from adjuvant therapies), the benefit from chemotherapy can be around 5% in the Case 1 and around 1% in Case 2

The magnitude of clinical benefit from chemotherapy varies by **clinical risk**

SPARANO ET AL. "CLINICAL AND GENOMIC RISK TO GUIDE THE USE OF ADJUVANT THERAPY FOR BREAST CANCER" NEJM 2019: 380: 2395-2405

ONCOTYPE DX ® AND PREDICT 2.1

	Oncotype DX ®	PREDICT 2.1
What is it?	A 21-gene assay	A prediction model based on patient databases
Validation	~10000 patients prospective study (TailorX)	Originally derived from ~2000 patients, independently validated in ~9000 patients
Uses	Tumor tissue	Age, menopausal status, detection method, estrogen receptor status, Her2 status, tumor size, grade, lymph node involvement, Ki67 (if available)
Results	Recurrence score (0-100).<br>No meaningful benefit from chemotherapy if age > 50 years with RS of 0-25 or ≤ 50 years with RS of 0-20	5, 10, 15-year overall survival<br>Overall survival benefit from chemotherapy, endocrine therapy, trastuzumab, bisphosphonates based on Early Breast Cancer Trialists Collaborative Group meta-analysis
Cost	CAD 4380	Free
Turnaround time	~ 2 weeks	Instant
Prognostic/ Predictive	Predictive > Prognostic	Prognostic > Predictive
Use	> 1 million patients	> 1 million patients
Limitations	Ignores clinical risk<br>Does not incorporate co-morbidities	Population level database, not individual risk<br>Does not incorporate co-morbidities

SPARANO ET AL. "CLINICAL AND GENOMIC RISK TO GUIDE THE USE OF ADJUVANT THERAPY FOR BREAST CANCER" NEJM 2019: 380: 2395-2405

TAILORX AND CLINICAL RISK

TailorX

- 9719 patients
- aged 18-75
- 0.6-5 cm tumors (0.6-1 with high risk features such as intermediate or poor nuclear/histological grade or lymphovascular invasion)
- Estrogen or progesterone positive
- Node negative (micrometastasis NOT included)

Originally planned to use adjuvant online for clinical risk but as no longer available used following definition of clinical risk:

- Low risk:
  - $\leq$ 1 cm and grade 3,
  - $\leq$ 2 cm and grade 2
  - $\leq$ 3 cm and grade 1
- If not low risk, all were considered high risk

Crude measure of clinical risk as ignores patient's age, menopausal status, ki67 if known, exact tumor size and detection method (screening or symptoms)

SPARANO ET AL. "CLINICAL AND GENOMIC RISK TO GUIDE THE USE OF ADJUVANT THERAPY FOR BREAST CANCER" NEJM 2019: 380: 2395-2405

CLINICAL RISK FROM TAILORX IN PATIENTS > 50 YEARS OF AGE

	% of total patients in TAILORX	Low clinical risk (%)	Low clinical risk estimated probability of distal recurrence at 9 years (%)	High clinical risk estimated probability of distal recurrence at 9 years
RS 0-10	12	76	2.6 +/- 0.8	7.4 +/- 3.4
RS 11-25 (no difference with addition of chemotherapy)	46	73	~3.8 +/- 0.6	~8.8 +/- 1.5
RS 26-100 (with chemotherapy)	10	43	7 +/- 2.4	19.8 +/- 3.9

Patients with low clinical risk have a favorable long-term prognosis

SPARANO ET AL. "CLINICAL AND GENOMIC RISK TO GUIDE THE USE OF ADJUVANT THERAPY FOR BREAST CANCER" NEJM 2019: 380: 2395-2405

CLINICAL RISK FROM TAILORX IN PATIENTS ≤ 50 YEARS OF AGE

	% of total patients in TAILORX	patients with RS who are low clinical risk (%)	Low clinical risk estimated probability of distal recurrence at 9 years (%)	High clinical risk estimated probability of distal recurrence at 9 years
RS 0-10	4.4	85	1.8 +/- 0.9	"0" only 64 patients
RS 11-25	23	76	~4.4 +/- 1.0	6.1 +/- 1.8 (with chemo)<br>12.3 +/- 2.4 (without chemo)
RS 26-100 (with chemotherapy)	4.3	43	6.2 +/- 2.5	15.2 +/- 3.3

<u>Patients with low clinical risk have a favorable long-term prognosis</u>

SPARANO ET AL. "CLINICAL AND GENOMIC RISK TO GUIDE THE USE OF ADJUVANT THERAPY FOR BREAST CANCER" NEJM 2019: 380: 2395-2405

ANOTHER GENOMIC TEST ASSESSING CLINICAL RISK

MINDACT, used Mammaprint ®, a 70-gene array to determine low or high genomic risk

Used clinical risk based on adjuvant online (no longer available) where low risk was defined as patients 10-year probability of breast cancer specific survival:

- > 88% breast cancer specific survival without systemic therapy if Estrogen receptor positive
- > 92% breast cancer specific survival without systemic therapy if Estrogen receptor negative
- All others high risk

Patients with low clinical risk and high genomic risk (n= 592) or vice versa (n=1550) had ~ 94-96% survival without distant metastasis at 5 year – **<u>excellent prognosis</u>**

No overall survival benefit from addition of chemotherapy in this discordant group. Possible small disease-free survival benefit (~3%) in high clinical risk and low genomic risk from chemotherapy.

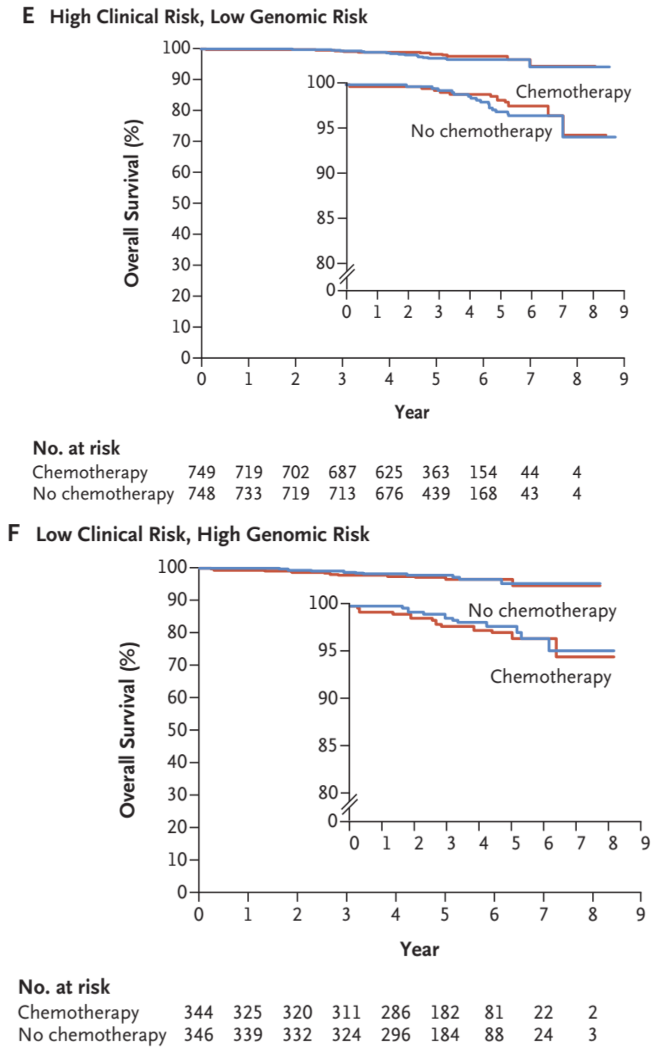

SPARANO ET AL. "CLINICAL AND GENOMIC RISK TO GUIDE THE USE OF ADJUVANT THERAPY FOR BREAST CANCER" NEJM 2019: 380: 2395-2405

CONCLUSION

Clinical risk determines magnitude of benefit from adjuvant chemotherapy.

Patients with low clinical risk have an excellent prognosis.

PREDICT 2.1 provides instant prognosis information with prediction of treatment benefit from meta-analysis at a population level

Oncotype DX ® provides predictive information based on individual patient's tumor but magnitude of benefit depends on clinical risk

Do patients with "low" clinical risk need a genomic test to determine benefit from adjuvant chemotherapy?

For the next 6 months you will be provided with PREDICT 2.1 results for all patients who would be potentially eligible for Oncotype DX ® testing.

SPARANO ET AL. "CLINICAL AND GENOMIC RISK TO GUIDE THE USE OF ADJUVANT THERAPY FOR BREAST CANCER" NEJM 2019: 380: 2395-2405
